# Rectal ectopic pregnancy: A case report

**DOI:** 10.1097/MD.0000000000024626

**Published:** 2021-02-12

**Authors:** Nguyen Manh Thang, Nguyen Thi Huyen Anh, Pham Hai Thanh

**Affiliations:** a Department of Obstetrics and Gynecology, Hanoi Medical University; b National Hospital of Obstetrics and Gynecology; c Textile and Garment Hospital, Hanoi, Vietnam.

**Keywords:** abdominal pregnancy, case report, laparoscopy, rectal, ultrasound

## Abstract

**Rationale:**

Abdominal ectopic pregnancy is a very rare form of ectopic pregnancy, yet is associated with higher morbidity due to atypical clinical presentation and misdiagnosis. In this report, we present a case of abdominal ectopic pregnancy with placenta invading to the rectal wall.

**Patient concerns:**

A 32-year-old woman was admitted to our hospital with an increasing serum ß-hCG level after diagnostic laparoscopy for ectopic pregnancy in the provincial hospital. During the laparoscopy, no gestational sac was found. She was discharged and scheduled for a follow-up visit to assess the level of ß-hCG. One week later, her serum ß-hCG level increased from 7000 IU/l to 12000 IU/l. Transvaginal Doppler ultrasound and abdominal computed tomography (CT) angiography demonstrated a right adnexal mass adherent to the rectal wall.

**Diagnosis:**

A rectal ectopic pregnancy is suspected.

**Interventions:**

Laparoscopic surgery was successfully performed in our hospital to remove the products of conception.

**Outcomes:**

Histologic examination confirmed the diagnosis of a rectal ectopic pregnancy. The patient had an uneventful recovery and was discharged the next few days.

**Lessons:**

This case report reveals that an abdominal pregnancy is remarkably difficult to diagnose and manage. The gynecologists need to be aware of the possibility of gestational sac between the uterus and the rectum. To make early diagnosis of abdominal pregnancy, they need to combine clinical findings, imaging techniques (ultrasound, CT, MRI) and serial human chorionic gonadotropin measurements. Laparoscopic management should be considered in early abdominal pregnancy. A multidisciplinary team of gynecologists and gastrointestinal surgeons is required to deal with rectal ectopic pregnancy.

## 1 Introduction

Ectopic pregnancy is the leading cause of maternal morbidity and mortality worldwide, accounting for 1.3% to 2% of all pregnancies.^[1,2]^ Ectopic pregnancy occurs when an embryo implants outside of the uterine cavity, usually in the Fallopian tubes (95%).^[3]^ Abdominal ectopic pregnancy is a very rare form of ectopic pregnancy, yet is associated with higher morbidity due to atypical clinical presentation and misdiagnosis.^[4,5,6]^ It is estimated to occur in ten per 100,000 pregnancies^[7]^ with a maternal mortality rate of approximately 2% to 30%.^[8]^ Abdominal ectopic pregnancy is also known as a life-threatening condition with risks of major hemorrhage, disseminated intravascular coagulation, bowel obstruction and fistulae.^[9]^

Several risk factors have been identified for ectopic pregnancy, including previous ectopic, previous tubal surgery, In Vitro Fertilization, pelvic infections, smoking, conception with the progesterone only pill or intrauterine device.^[10]^ Risk factors for abdominal pregnancy are common with those for tubal pregnancy.^[11,12]^ Patients with ectopic pregnancy normally present with abdominal pain and vaginal bleeding during the first trimester.^[13]^ However, some women with ectopic pregnancy are asymptomatic with complaints of diarrhea, dizziness or vomiting.^[14]^

Currently, diagnosis of ectopic pregnancy is established based on a combination of transvaginal ultrasonography and measurement of serum ß-hCG concentrations. For the diagnosis of abdominal pregnancy, abdominal X-ray, computed tomography, magnetic resonance imaging, and diagnostic laparoscopy are commonly utilized. There is little data on the optimal treatment strategies for abdominal pregnancies; however, surgical management is still considered as the mainstay of treatment.^[15]^

In this report, we describe an unique case of abdominal ectopic pregnancy with placenta invading to the rectal wall.

## 2 Case report

A 32-year-old woman, gravida 1, para 1, who had a caesarean delivery 6 years ago, was admitted to our hospital with an increasing serum ß-hCG level after conservative laparoscopic surgery for ectopic pregnancy. At first, she was referred to the provincial hospital for evaluation of spotting and mild abdominal pain at 6 weeks of gestation. Her serum ß-hCG level was 7000 IU/l, and transvaginal sonography showed an empty uterine cavity. She was diagnosed with ectopic pregnancy and underwent a diagnostic laparoscopy. At laparoscopy, 2 ovaries appeared normal and the infundibulum of left tube had light bleeding. A small amount of free fluid was seen in the pelvis. No trophoblastic tissue was found in the abdominal cavity. The patient was discharged from the provincial hospital on postoperative day 3. At a scheduled visit 1 week later, her serum ß-hCG level increased to 12000 IU/l. Soon after, she was transferred to our hospital.

**Figure 1:**
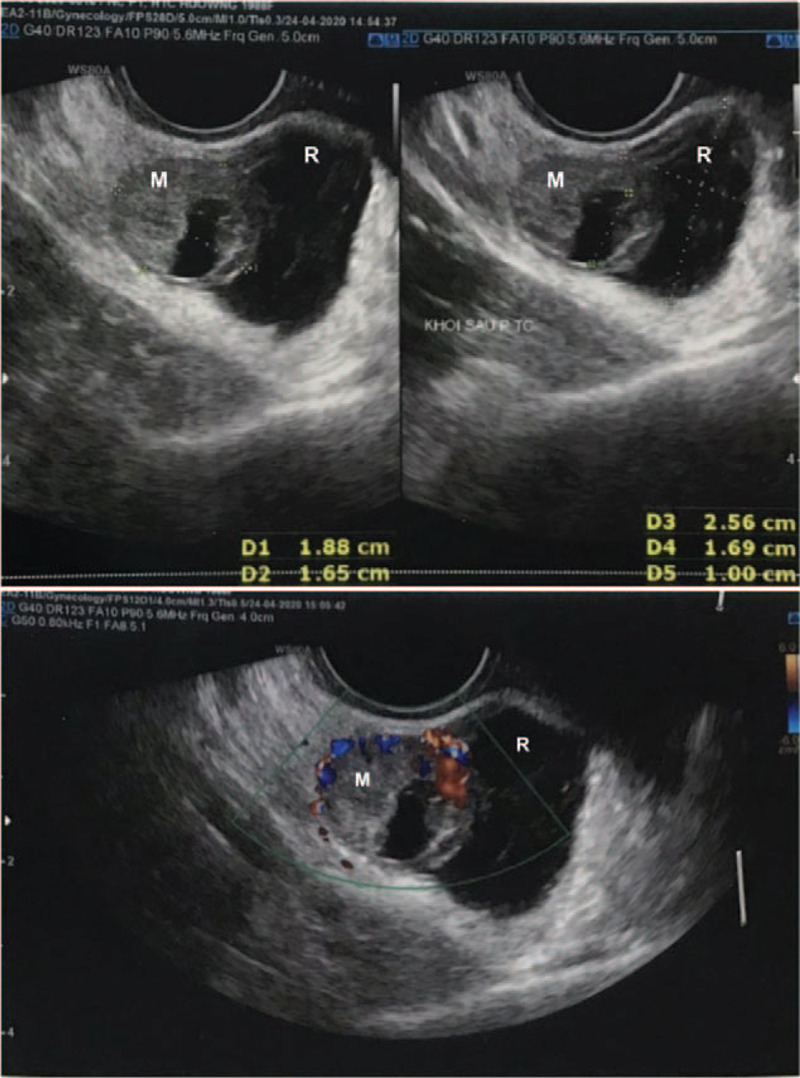
Transvaginal Doppler ultrasound showing a 19 × 17 mm mass with a yolk sac on the day of admission (M: mass, R: rectum).

**Figure 2:**
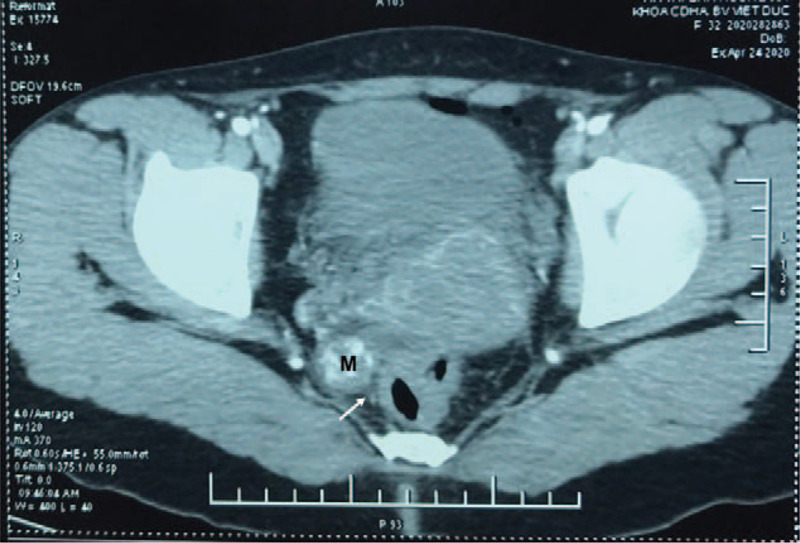
Abdominal CT angiography on the day of admission (M: mass, arrow: mass in contact with the rectal wall).

On the day of admission, a physical examination revealed some vaginal discharge, but no vaginal bleeding. The abdominal examination was unremarkable. Her serum ß-hCG level rose to 16088 IU/l on postopereative day 12. Transvaginal Doppler ultrasound showed a 19 × 17 mm mass that contained a yolk sac and located in the right of rectum (Fig. 1). A heterogeneous hypoechoic mass measuring 26 × 17 mm was close to this mass (Fig. 1). No gestational sac was visualized in the uterine cavity. Abdominal Computed tomography (CT) angiography was then performed to determine the location of her adnexal mass. It demonstrated a right adnexal mass measuring 20 × 20 mm adherent to the rectal wall (Fig. 2). No thickening of the rectal wall was seen.

On postoperative day 14, another laparoscopic surgery was performed. No blood was detected in the peritoneal cavity. Both ovaries and Fallopian tubes looked normal. A 3 × 3 cm gestational sac coated with blood clots was found behind the uterus in the right pouch of Douglas. The placenta tissue was identified to implant on the anterior wall of the rectum. Removal of the placenta revealed a 1 cm size defect in the rectal wall. A gastrointestinal surgeon was involved to investigate and suture the rectal mucosa. Hemostasis was achieved with electrocautery and surgicel. Two days after the surgery, her serum ß-hCG level decreased from 12000 IU/l to 3700 IU/l.

Histologic examination of the surgical specimens confirmed the diagnosis of a rectal ectopic pregnancy with direct invasion of trophoblastic tissue into the rectal wall. The patient made an uneventful recovery and was discharged from hospital on postoperative day 20. At a scheduled visit 2 months later, her serum ß-hCG level decreased to less than 5 IU/l.

## 3 Discussion

Abdominal ectopic pregnancy is a rare and unusual condition. The majority of these cases are secondary and result from re-implantation of a tubal rupture or a tubo-abdominal abortion.^[16]^ The present reported case is considered to be a primary abdominal pregnancy, which meets the diagnostic criteria described by Studdiford in 1942.^[17]^ These include:

1. Normal tubes and ovaries with no evidence of recent or remote injury.
2. An absence of any evidence of an utero-peritoneal fistula.
3. The presence of a pregnancy related exclusively to the peritoneal surface and early enough to eliminate the possibility of secondary implantation following a primary nidation in the tube.

Most of abdominal pregnancy are in recto-unterine and vesico-uterine pouchs, with placental attachment to the rectal or uterine serosa.^[18]^ The clinical presentation of abdominal ectopic pregnancy can be similar to tubal ectopic pregnancy, with amenorrhea, lower abdominal pain and vaginal bleeding.^[19,20]^ Some reported cases described rectal bleeding as an complication of rectal ectopic pregnancy.^[21,22]^ However, we were completely unaware of this complication in our patient.

Abdominal ectopic pregnancy is frequently misdiagnosed. An early diagnosis of this condition is significant to minimize the risk of maternal morbidity and mortality. Ultrasound remains the first-line method for diagnosing abdominal pregnancy; however, its sensitivity is inconsistent, ranging from 50% to 90%.^[23]^ The Royal college of Obstetricians and Gynecologists^[24]^ recommend the use of ultrasound criteria suggested by Gerli et al^[25]^ to diagnose an abdominal ectopic pregnancy. These include:

1. Absence of an intrauterine gestational sac.
2. Absence of both a clearly dilated tube and a complex adnexal mass.
3. A gestational cavity surrounded by loops of bowel and separated by peritoneum.
4. A wide mobility similar to fluctuation of the sac, particularly evident with a gentle pressure of the transvaginal probe toward the posterior cul-de-sac.

Magnetic resonance imaging can be a useful diagnostic tool in advanced abdominal pregnancy and can help to plan the surgical management.^[24]^ MRI helps determine the accurate localization of the placenta, detect the arterial feeders, and assess the placental adherence to surrounding organs.^[26]^ Computed tomography is also helpful to confirm the diagnosis of abdominal pregnancy.^[27]^ In our hospital, CT is commonly used instead of MRI because CT examination is less time consuming and cheaper.

Serial serum ß-hCG measurements are also necessary for a pregnancy of unknown location or when abdominal pregnancy is suspected. In our case, patient underwent the first laparoscopic surgery in the provincial hospital. During the laparoscopy, no ectopic pregnancy was found. However, her serum ß-hCG level nearly doubled at a scheduled visit 1 week later. When she was transferred to our hospital, the increase of serum ß-hCG level and imaging findings suggested the diagnosis of an abdominal pregnancy. We performed the second laparoscopy and detected a gestational sac in Douglas’ pouch with the placenta tissue implanted on the rectal wall.

Laparoscopic surgery is an option for treatment of early abdominal pregnancy.^[24]^ Advanced abdominal pregnancy should be managed by laparotomy which allows better access to handle placental adherence and control the hemorrhage.^[24,28,29]^ In the present case, diagnosis was established in early pregnancy (<12 weeks) and the implantation site allows a non-hemorrhagic surgical excision (rectal wall); therefore, laparoscopy was chosen instead of laparotomy to remove the products of conception.

## 4 Conclusion

Our case report illustrates that an abdominal pregnancy, especially rectal pregnancy, is remarkably difficult to diagnose and manage. Hence, the gynecologists need to be aware of the possibility of gestational sac between the uterus and the rectum, particularly when an ectopic pregnancy is suspected but no tubal pregnancy is detected in diagnostic laparoscopy. To make early diagnosis of abdominal pregnancy, they need to combine clinical findings, imaging techniques (ultrasound, CT, MRI) and serial human chorionic gonadotropin measurements. With regard to treatment, laparoscopic management should be considered in early abdominal pregnancy. In case of rectal pregnancy, a multidisciplinary team of gynecologists and gastrointestinal surgeons is required to deal with placental adherence and rectal injuries.

## Author contributions

**ANTH:** Writing – Original Draft, Visualization.

**TNM, ANTH, TPH:** Conceptualization, Methodology.

**TNM:** Supervision, Writing – Review & Editing.

The final manuscript was approved by all authors.

## Abbreviations

Abbreviations: CT = computed tomography, MRI = magnetic resonance imaging, ß-hCG = ß-human chorionic gonadotropin.
